# Long-Term Lesion Progression After Left Main Distal Bifurcation Stenting: Insights From Bifurcation Angle Variation Throughout the Cardiac Cycle

**DOI:** 10.31083/RCM45495

**Published:** 2026-05-13

**Authors:** En Chen, Danqing Hu, Hong Zheng, Long Chen, Mingming Hu, Lianglong Chen, Wei Cai

**Affiliations:** ^1^Department of Cardiology, Fujian Medical University Union Hospital, Fujian Medical University, 350001 Fuzhou, Fujian, China; ^2^Fujian Heart Medical Center, Fujian Medical University Union Hospital, Fujian Medical University, 350001 Fuzhou, Fujian, China; ^3^Fujian Institute of Coronary Artery Disease, Fujian Medical University Union Hospital, Fujian Medical University, 350001 Fuzhou, Fujian, China; ^4^School of Health, Fujian Medical University, 350122 Fuzhou, Fujian, China; ^5^Shanghai Pulse Medical Technology, Inc, 200030 Shanghai, China

**Keywords:** coronary artery disease, anatomy, cardiac cycle, disease progression, stents

## Abstract

**Background:**

The clinical impact of changes in the bifurcation angle throughout the cardiac cycle (BA_C_) after percutaneous coronary intervention (PCI) for left main coronary bifurcation lesions (LMCBLs) remains controversial, and the associated long-term evolution post-stenting remains unknown. Therefore, this study aimed to evaluate temporal changes in the BA_C_ and the related impact on lesion progression in patients undergoing single- or dual-stenting.

**Methods:**

Proximal (PBA_C_) and distal (DBA_C_) bifurcation angles were quantified throughout the cardiac cycle using two-dimensional quantitative coronary angiography at optimal views before the procedure, immediately after, and at long-term follow-up. These measurements represented the absolute difference between the end-diastolic and end-systolic angles for the left main (LM) to the left circumflex (LCX) and for the left anterior descending (LAD) to the LCX. Lesion progression was assessed from increases in diameter stenosis percentage (iDS%) from post-procedure to follow-up.

**Results:**

A total of 284 patients underwent single-stenting (LM-LAD), and 84 underwent dual stenting (LM-LAD-LCX). Changes in the PBA_C_ were unaffected by interventional strategies or time. The DBA_C_ was narrowed post-stenting in all patients, but rebounded to pre-procedural levels during follow-up in the single-stenting group. In contrast, the DBA_C_ remained at post-procedural levels in the dual-stenting group. Lesion progression was more pronounced in patients with dual stenting, particularly in the LCX. The pre-procedural PBA_C_ correlated linearly with the iDS%-LCX metric in the dual stenting.

**Conclusions:**

The PBA_C_ remained stable over time and across strategies, whereas the DBA_C_ decreased post-stenting. During follow-up, the DBA_C_ rebounded in the single-stenting group but remained low in the dual-stenting group. The pre-procedural PBA_C_ represents an independent anatomical risk marker for future LCX progression in patients with dual-stented LMCBLs.

## 1. Introduction

Percutaneous coronary intervention (PCI) for left main coronary bifurcation lesion (LMCBL) is becoming increasingly common, as has been proven to be a reasonable option to coronary artery bypass grafting (CABG) surgery, especially in patients with a lower Syntax score [1, 2]. However, it remains inferior to CABG in the interventional treatment of complex LMCBL, primarily due to a higher rate of target lesion revascularization [3, 4]. Part of this challenge may stem from limitations in the angiographic classification systems used for procedural planning. The Medina classification, while widely adopted, does not incorporate the bifurcation angle (BA)—a fundamental anatomical characteristic. This omission limits its utility for comparing complex lesions and may contribute to clinical ambiguity and research stagnation [5].

Recognizing this gap, the Movahed classification was proposed as a more anatomically descriptive system that specifically includes the BA [6]. The development of this angle-inclusive classification underscores a growing consensus on the physiological importance of the BA. Indeed, substantial evidence links both the static BA and its change throughout the cardiac cycle (BA_C_) in LMCBL to an increased risk of restenosis, particularly after dual stenting [7, 8, 9, 10, 11]. Nevertheless, the precise prognostic value of BA/BA_C_ remains inconsistent. For instance, a substudy of the SYNTAX trial found that post-procedural systolic-diastolic distal BA (between left anterior descending (LAD) and left circumflex (LCX)) was associated with higher 5-year event rates [12], whereas pre-procedural distal BA showed no significant correlation with outcomes at 12 months or 5 years [12, 13]. These highlight a key gap: the longitudinal evolution of BA_C_ after stenting and its relationship with lesion progression are poorly understood.

Consequently, we performed this study to determine the temporal changes in BA_C_ (pre-procedure, post-procedure and long-term follow-up) in LMCBL patients, who underwent PCI using either a single stenting or dual stenting technique, and the relation between BA_C_ and lesion progression.

## 2. Materials and Methods

### 2.1 Patients Selection

This retrospective study was conducted at Fujian Medical University Union Hospital between January 2013 and December 2021. We consecutively enrolled patients with LMCBL who had undergone PCI and had completed follow-up coronary angiography at a minimum of 6 months. Eligible patients needed to have angiograms available for BA_C_ analysis at three time points: pre-procedure, immediately post-procedure, and at long-term follow-up, covering both left main (LM) to LAD single stenting and LM-LAD-LCX dual stenting techniques. Key exclusion criteria were: (1) a history of CABG; (2) an excessively short LM length (<3 mm) precluding reliable BA_C_ measurement; (3) total occlusion of the proximal LAD or LCX within 10 mm distal to the bifurcation core; and (4) significant vessel overlap at the LMCBL segment that would compromise the quality of separate angiographic assessments. Clinical characteristics, laboratory findings, and procedural details were collected for all included patients.

### 2.2 Angiographic Analysis and Study Definition

In view of the disparity between the three-dimensional (3D) angiographic reconstruction and the actual coronary artery in LMCBL, and given that our study’s target variable is the range of BA between diastole and systole in LMCBL patients over time, our study performed the BA_C_ analysis from the two-dimensional (2D) optimal view of left main bifurcation angiogram at the three specified time points, maintaining consistent optimal views throughout (pre-procedure, post-procedure, and long-term follow-up). Experienced analysts conducted BA_C_ and quantitative coronary angiography (QCA) measurements of the LMCBL from these optimal angiograms at the aforementioned three time points using AngioPlus software (Ver. 2.0, Pulse Medical Imaging Technology, Shanghai, China).

The BA in LMCBL was presented in accordance with the European Bifurcation Club consensus [14]. The proximal bifurcation angle (PBA) in LMCBL was defined as the angle between LM and LCX, while the distal bifurcation angle (DBA) in LMCBL was delineated between LAD and LCX. Angles at end-diastole and end-systole, both before and immediately after the interventional procedure, as well as during long-term follow-up, were concurrently evaluated. The ranges of PBA and DBA throughout the cardiac cycle were expressed as PBA_C_ and DBA_C_, representing the absolute difference between the end-diastolic and end-systolic angle values.

Long-term lesion progression was determined by the change in percent diameter stenosis (DS%) from post-procedure to long-term follow-up. This was expressed as an increase in DS% (iDS%) across three segments of the LMCBL.

### 2.3 Statistical Methods 

All statistical analyses were performed with SPSS (version 24; SPSS Inc, Chicago, IL, USA). Continuous variables were tested for normality using the Shapiro-Wilk test and were presented as mean ± standard deviation or median (interquartile range), as appropriate. Categorical variables were expressed as numbers (percentages), with comparisons made using the Pearson chi-square test. To evaluate temporal changes in PBA_C_, DBA_C_, and iDS% across the three LMCBL segments at the three time points (pre-procedure, post-procedure, long-term follow-up), the matched Friedman test was employed. Differences in BA_C_ and iDS% when stratified by interventional strategy (single or dual stenting for LMCBL) were assessed using the independent Mann-Whitney U test. Comparisons of continuous variables between two groups that met assumptions of normality and homogeneity of variance were conducted using the independent samples *t*-test. Statistical significance was set at a two-sided *p*-value of less than 0.05.

The reproducibility of pre-procedural bifurcation angle measurements was assessed by evaluating both intra- and inter-observer variability. A randomly selected sample of 40 patients (20 from each stenting group) was used for this analysis. Measurements were performed by the primary observer and an independent observer following standardized procedures. Reliability was quantified using the Intraclass Correlation Coefficient (ICC) with a two-way random-effects model for absolute agreement.

Hierarchical multiple linear regression was performed to investigate whether BA_C_ was a significant predictor for iDS%, after accounting for the predictive contribution of previously identified arteriosclerosis-related demographic and interventional variables. The regression model consisted of three separate blocks of variables, with categorical variables being transformed into dummy variables. In the regression model, age, gender, the time of follow-up, body mass index (BMI), diabetes mellitus, hypertension, current smoking, dyslipidemia, low-density lipoprotein cholesterol (LDL-C), left ventricular ejection fraction (LVEF), the use of intravascular ultrasound (IVUS) or optical coherence tomography (OCT), and the DS% immediately post-procedure were entered in the first block. Interventional strategies, including single stenting and dual stenting in LMCBL, were entered in the second block. Next, BA_C_ (PBA_C_ and DBA_C_) measured before and immediately after the procedure were entered in the third block. The R^2^ changed for each block was tested.

The analytical framework of this study distinguished between descriptive comparisons and the core, pre-specified inferential analyses. Comparisons of baseline and follow-up characteristics were primarily descriptive and exploratory, whereas the study’s main conclusions were based strictly on the hierarchical multiple regression and associated correlation analyses.

## 3. Results

### 3.1 Baseline Characteristics 

A total of 368 LMCBL patients were included in this study, stratified according to the interventional strategies, with 284 patients undergoing PCI using a single stent from the LM to the LAD (single stenting group), and 84 patients undergoing PCI with dual stenting in the LM-LAD-LCX (dual stenting group). There were no significant differences in baseline characteristics between the two groups, except for the percentage of dyslipidemia, as shown in Table 1. Second-generation drug-eluting stents were implanted in all included patients. The population was predominantly male (299 patients, 81.3%) and had a mean age of 65.3 ± 9.8 years. The median follow-up duration was 379 days.

**Table 1.** **Demographic and clinical characteristics of the study cohort**.

	All patients (n = 368)	Single stenting (n = 284)	Dual stenting (n = 84)	*p* value
Age at baseline, years	65.3 ± 9.8	65.3 ± 9.9	65.6 ± 9.5	0.820
Male gender, n (%)	299 (81.3%)	232 (81.7%)	67 (79.8%)	0.691
BMI, kg/m^2^	24.2 (22.1–26)	24.2 (22.1–26.2)	24.1 (22.1–25.4)	0.393
Prior myocardial infarction, n (%)	58 (15.8%)	43 (15.1%)	15 (17.9%)	0.548
Diabetes mellitus, n (%)	133 (36.1%)	109 (38.4%)	24 (28.6%)	0.100
Hypertension, n (%)	242 (65.8%)	189 (66.5%)	53 (63.1%)	0.558
Dyslipidemia, n (%)	132 (35.9%)	110 (38.7%)	22 (26.2%)	0.035
Current smoking, n (%)	127 (34.5%)	99 (34.9%)	28 (33.3%)	0.796
Total cholesterol, mmol/L	3.9 (3.3–4.9)	4 (3.3–4.9)	3.9 (3.4–4.6)	0.999
LDL-C, mmol/L	2.5 (2–3.3)	2.5 (2–3.4)	2.4 (2–3.1)	0.751
HDL-C, mmol/L	1 (0.8–1.1)	1 (0.8–1.1)	1 (0.9–1.1)	0.490
Serum creatinine, μmol/L	80 (69–93.8)	80 (70–94)	78.5 (67–92.8)	0.369
LVEF, %	64 (56.3–68.8)	63 (56.1–68.7)	65.3 (57.4–69.6)	0.203
Follow-up, days	379 (361–439.8)	381 (361–461.3)	378 (359.3–419.3)	0.407
Intermediate coronary artery, n (%)	51 (13.9%)	46 (16.2%)	5 (6%)	0.017
IVUS/OCT, n (%)	115 (31.3%)	91 (32%)	24 (28.6%)	0.547
Rotational Atherectomy, n (%)	18 (4.9%)	13 (4.6%)	5 (6%)	0.822

Values were n (%), mean ± standard deviation, and median (interquartile range). BMI, body mass index; LDL-C, low-density lipoprotein cholesterol; HDL-C, high-density lipoprotein cholesterol; LVEF, left ventricular ejection fraction; IVUS/OCT, intravascular ultrasound/optical coherence tomography.

The morphologies observed in LMCBL were not comparable, with fewer occurrences of the intermediate coronary artery in the dual stenting group. However, the usage of intracoronary imaging devices (IVUS/OCT) was similar between groups, as was the incidence of severe calcification lesions requiring rotational atherectomy.

### 3.2 Assessments of BA_C_

**Fig. 1.**
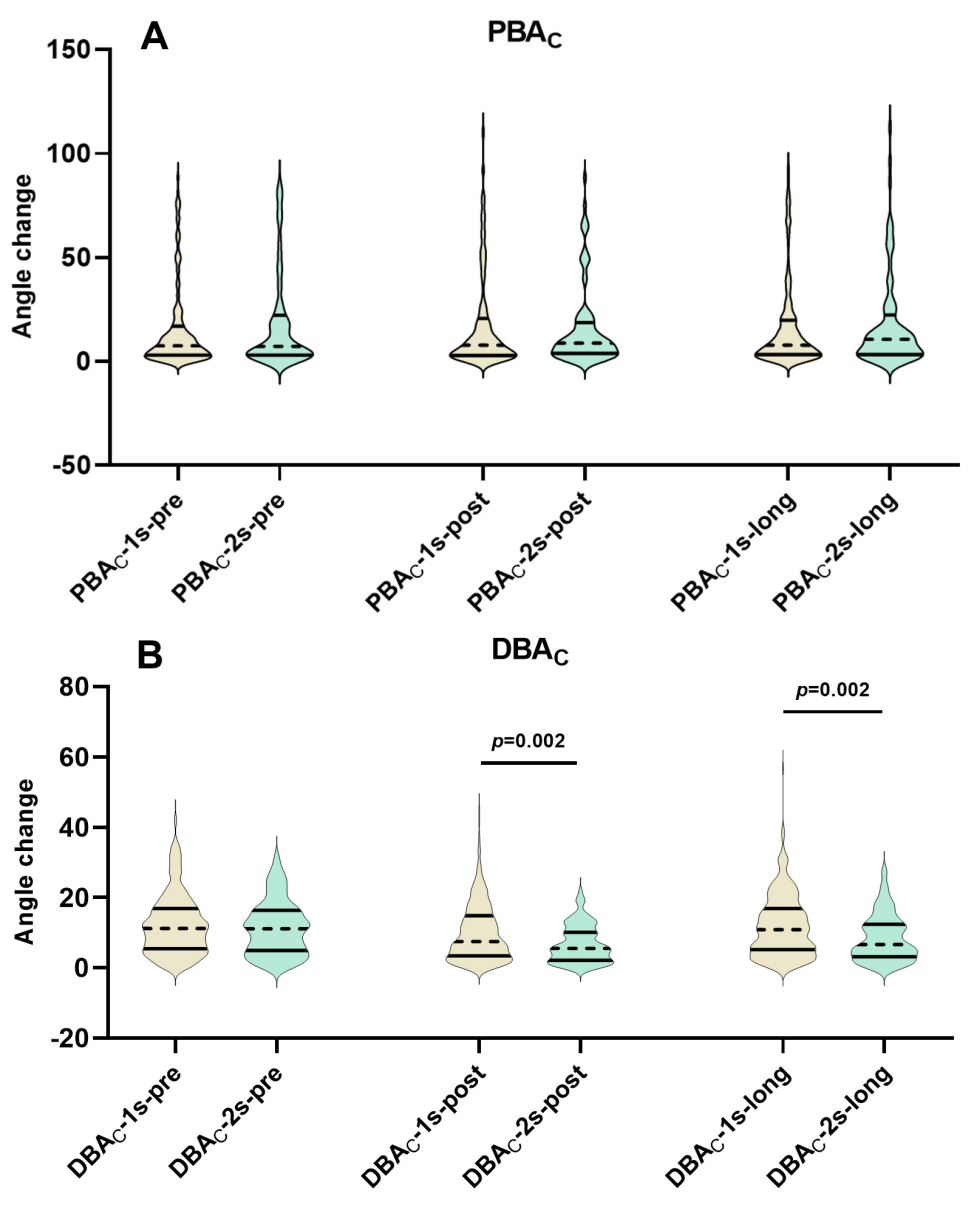
**Temporal changes in PBA_C_ and DBA_C_ following single versus dual stenting**. (A) Comparison of PBA_C_ among pre-procedure, post-procedure and long-term follow-up assessments between the single and dual stenting strategies. (B) Comparison of DBA_C_ among pre-procedure, post-procedure and long-term follow-up assessments between the single and dual stenting strategies. PBA_C_, proximal bifurcation angle change throughout the cardiac cycle; DBA_C_, distal bifurcation angle change throughout the cardiac cycle; PBA_C_/DBA_C_-1s-pre, PBA_C_/DBA_C_-1s-post and PBA_C_/DBA_C_-1s-long, PBA_C_/DBA_C_ pre-procedure, post-procedure, and at long-term follow-up in distal left main bifurcation patients treated with single stenting; PBA_C_/DBA_C_-2s-pre, PBA_C_/DBA_C_-2s-post and PBA_C_/DBA_C_-2s-long, PBA_C_/DBA_C_ pre-procedure, post-procedure, and at long-term follow-up in distal left main bifurcation patients treated with dual stenting.

**Fig. 2.**
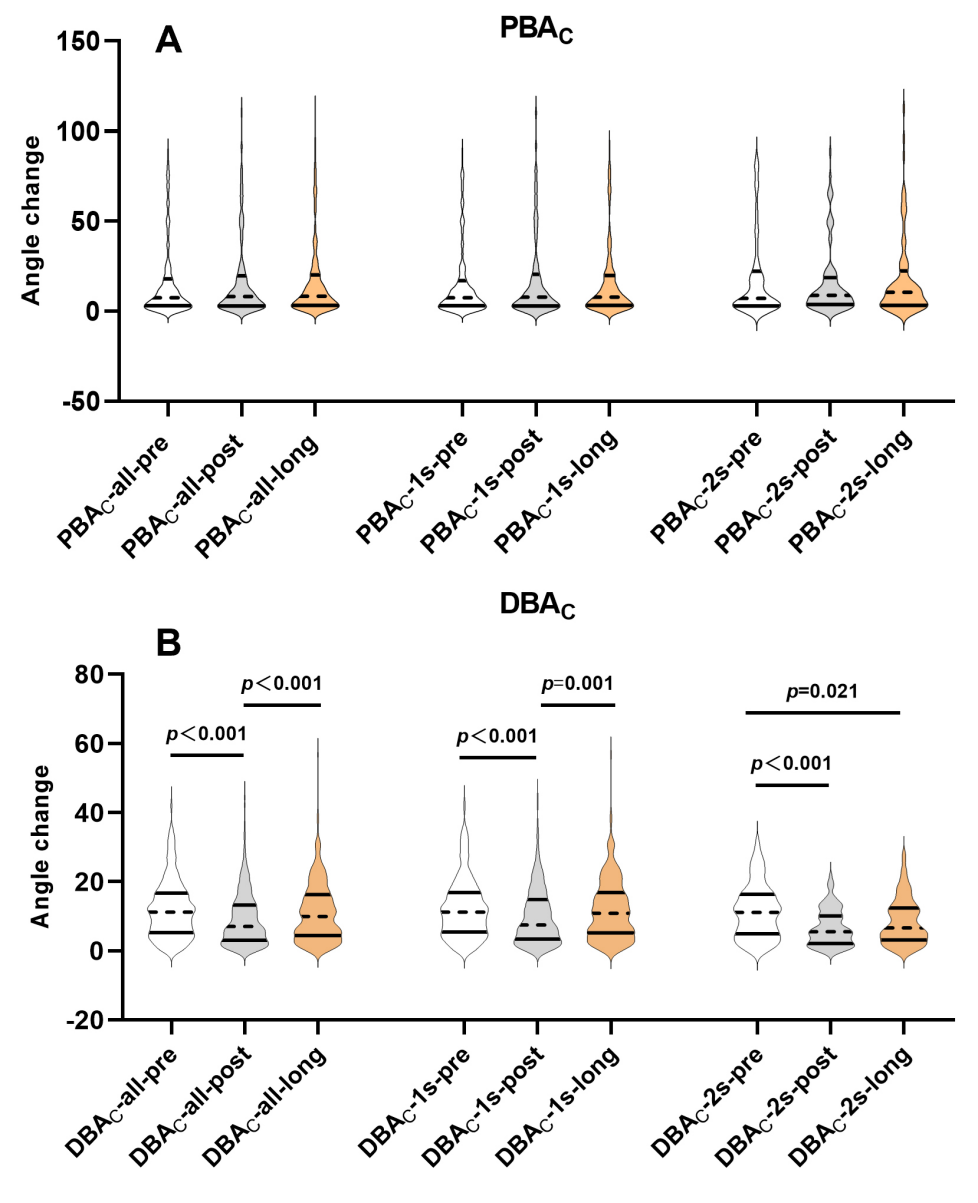
**Temporal trends of PBA_C_ and DBA_C_ within single versus dual stenting groups**. (A) PBA_C_ compared across pre-procedural, post-procedural, and long-term follow-up time points within the single stenting group and separately within the dual stenting group. (B) DBA_C_ compared across pre-procedural, post-procedural, and long-term follow-up time points within the single stenting group and separately within the dual stenting group. PBA_C_ compared across pre-procedural, post-procedural, and long-term follow-up time points within the single stenting group and separately within the dual stenting group. PBA_C_, proximal bifurcation angle change throughout the cardiac cycle; DBA_C_, distal bifurcation angle change throughout the cardiac cycle; LMCBL, left main coronary bifurcation lesion; PBA_C_/DBA_C_-all-pre, PBA_C_/DBA_C_-all-post and PBA_C_/DBA_C_-all-long, PBA_C_/DBA_C_ pre-procedure, post-procedure, and at long-term follow-up in all included patients; PBA_C_/DBA_C_-1s-pre, PBA_C_/DBA_C_-1s-post and PBA_C_/DBA_C_-1s-long, PBA_C_/DBA_C_ pre-procedure, post-procedure, and at long-term follow-up in distal left main bifurcation patients treated with single stenting; PBA_C_/DBA_C_-2s-pre, PBA_C_/DBA_C_-2s-post and PBA_C_/DBA_C_-2s-long, PBA_C_/DBA_C_ pre-procedure, post-procedure, and at long-term follow-up in distal left main bifurcation patients treated with dual stenting.

The analysis demonstrated excellent reproducibility for BA_C_ measurements. The intra-observer ICC was 0.99 (95% CI: 0.99–1.00) for pre-procedural PBA_C_ and 0.97 (95% CI: 0.94–0.98) for pre-procedural DBA_C_. The inter-observer ICC was 0.99 (95% CI: 0.99–1.00) for PBA_C_ and 0.98 (95% CI: 0.97–0.99) for pre-procedural DBA_C_. The details of PBA_C_ and DBA_C_ between the two groups were shown in Tables 2,3. Regarding PBA_C_, whether before procedure (PBA_C_-pre), post-procedure (PBA_C_-post), or during long-term follow-up (PBA_C_-long), there were no differences in terms of single or dual stenting in LMCBL, shown in Fig. 1A. The PBA_C_ in the LMCBL using the single stenting technique, as well as the LMCBL with the dual stenting technique, were not subject to changes due to stent implantation and over time, as indicated in Fig. 2A.

**Table 2.** **Bifurcation angle and angiographic lesions characteristics**.

	All patients (n = 368)	Single stenting (n = 284)	Dual stenting (n = 84)	*p* value
Diastolic PBA-pre	141.8 (102.3–165.5)	143.1 (103–165.8)	136.1 (94.7–164.5)	0.408
Systolic PBA-pre	136.6 (106.4–162.1)	137.4 (105.4–162.7)	134.4 (108.6–159.2)	0.929
PBA_C_-pre	7.4 (3.1–18.1)	7.5 (3.1–17)	7.2 (3–22.2)	0.602
Diastolic PBA-post	126 (99.4–166)	131.6 (101.7–166.9)	113.6 (94.4–156.4)	0.073
Systolic PBA-post	126.6 (106.4–163.3)	130.9 (107.9–164.3)	118.4 (103.8–155.1)	0.159
PBA_C_-post	8.2 (3–19.7)	7.8 (2.9–20.6)	8.8 (3.8–18.7)	0.720
Diastolic PBA-long	130.3 (101.1–162.8)	131 (101–165.12)	127.6 (101–157.8)	0.455
Systolic PBA-long	130.9 (110.1–163.3)	132.8 (110.7–164.1)	123.4 (106.9–158.2)	0.091
PBA_C_-long	8.4 (3.3–20.2)	7.8 (3.3–19.8)	10.6 (3.3–22.3)	0.553
Diastolic DBA-pre	85.8 (68.8–101.4)	86.1 (71–102.8)	81.5 (63.3–96.8)	0.029
Systolic DBA-pre	74.7 (60.7–90.2)	75.5 (61.7–91.8)	69.3 (56.2–87.3)	0.028
DBA_C_-pre	11.2 (5.3–16.7)	11.2 (5.5–16.9)	11.1 (4.9–16.4)	0.462
Diastolic DBA-post	81.3 (68.3–97.7)	83.8 (70.9–100.3)	73.8 (59.6–85.7)	<0.001
Systolic DBA-post	73.6 (62.6–88.9)	75.8 (63.5–90.5)	70.1 (55.7–79.6)	<0.001
DBA_C_-post	7.1 (3.1–13.2)	7.5 (3.4–14.8)	5.6 (2.1–10.1)	0.002
Diastolic DBA-long	79 (67.6–96.6)	82.8 (69.9–98.8)	73 (57.7–84.2)	<0.001
Systolic DBA-long	70.6 (59.8–83.1)	72.1 (61.6–84.7)	65 (56.6–77.3)	<0.001
DBA_C_-long	9.9 (4.4–16.3)	10.8 (5.2–16.9)	6.7 (3.2–12.4)	0.002
DS%-pre in LM	48.8 (28.1–64.1)	47.3 (26.3–62.1)	54 (34–69.7)	0.004
DS%-pre in LAD	62.8 (54.1–68.9)	62.4 (53.7–68.6)	63.8 (54.8–70.2)	0.495
DS%-pre in LCX	25.4 (16.4–46.1)	20.4 (13.9–29.6)	54.5 (46.2–60.8)	<0.001
DS%-post in LM	2.8 (0–5.5)	3 (0–5.7)	2.2 (0–4.9)	0.086
DS%-post in LAD	5.9 (3.2–8.8)	5.7 (3.1–8.7)	6.7 (3.6–10.1)	0.179
DS%-post in LCX	22.7 (14.9–32)	25.8 (19.6–34.5)	10.7 (5.8–16.3)	<0.001
DS%-long in LM	7 (4.9–11.1)	6.8 (4.7–10.9)	7.9 (5.3–11.7)	0.217
DS%-long in LAD	10.2 (6.4–15.9)	9.6 (6–14.9)	12.2 (7.4–18.1)	0.008
DS%-long in LCX	30.7 (21.9–40.9)	31.6 (22.7–40.7)	26 (17–43.9)	0.040
iDS%-LM	3.4 (1.2–6.9)	3.1 (1.1–6.5)	4.7 (1.9–8.8)	0.013
iDS%-LAD	2.9 (1.3–7.3)	2.8 (1.2–6.5)	4.2 (1.6–9.2)	0.016
iDS%-LCX	2.6 (0.9–12.6)	2.2 (0.7–6.5)	15.6 (2.5–28.6)	<0.001

Note: *p*-values were for descriptive/exploratory purposes only, not for inferential testing of causality or efficacy. The study’s primary conclusions were based on pre-specified regression and correlation analyses. Values were median (interquartile range). PBA, proximal bifurcation angle; DBA, distal bifurcation angle; PBA_C_, PBA change throughout the cardiac cycle; DBA_C_, DBA change throughout the cardiac cycle; PBA/DBA-pre, PBA/DBA-post and PBA/DBA-long, PBA/DBA pre-procedure, post-procedure, and at long-term follow-up; PBA_C_/DBA_C_-pre, PBA_C_/DBA_C_-post and PBA_C_/DBA_C_-long, PBA_C_/DBA_C_pre-procedure, post-procedure, and at long-term follow-up; DS%, percent diameter stenosis; iDS%, increase in DS%; DS%-pre, DS%-post and DS%-long, DS% pre-procedure, post-procedure, and at long-term follow-up; LM, left main; LAD, left anterior descending; LCX, left circumflex; iDS%-LM, iDS%-LAD, and iDS%-LCX, iDS% in LM, LAD and LCX.

**Table 3.** **Temporal changes in PBA_C_ and DBA_C_**.

	Before procedure	Post procedure	Long-term follow-up	*p* value
PBA_C_-all	7.4 (3.1–18.1)	8.2 (3–19.7)	8.4 (3.3–20.2)	0.453
PBA_C_-single stenting	7.5 (3.1–17)	7.8 (2.9–20.6)	7.8 (3.3–19.8)	0.374
PBA_C_-dual stenting	7.2 (3–22.2)	8.8 (3.8–18.7)	10.6 (3.3–22.3)	0.965
DBA_C_-all	11.2 (5.3–16.7)	7.1 (3.1–13.2)	9.9 (4.4–16.3)	<0.001
DBA_C_-single stenting	11.2 (5.5–16.9)	7.5 (3.4–14.8)	10.8 (5.2–16.9)	<0.001
DBA_C_-dual stenting	11.1 (4.9–16.4)	5.6 (2.1–10.1)	6.7 (3.2–12.4)	<0.001

Note: *p*-values were for descriptive/exploratory purposes only, not for inferential testing of causality or efficacy. The study’s primary conclusions were based on pre-specified regression and correlation analyses. Values were median (interquartile range). PBA_C_, proximal bifurcation angle change throughout the cardiac cycle; DBA_C_, distal bifurcation angle change throughout the cardiac cycle; PBA_C_/DBA_C_-all, PBA_C_/DBA_C_ in all included patients; PBA_C_/DBA_C_-single stenting, PBA_C_/DBA_C_ in the single stenting group; PBA_C_/DBA_C_-dual stenting, PBA_C_/DBA_C_ in the dual stenting group.

DBA_C_ changes over time were displayed in Table 3 and Fig. 2B, indicating a tendency for DBA_C_ to narrow immediately post-stenting and subsequently widen over time. This change was mainly observed in the single stenting group, which exhibited similar DBA_C_-pre and DBA_C_-long values but lower DBA_C_-post values (pre-procedure 11.2 (5.5–16.9) vs. post-procedure 7.5 (3.4–14.8) vs. long-term follow-up 10.8 (5.2–16.9), *p* < 0.001). In contrast, although the dual stents group also exhibited a narrower DBA_C_ post-procedure (post-procedure 5.6 (2.2–10.1) vs. pre-procedure 11.1 (4.9–16.4), *p* < 0.001), the long-term DBA_C_ values remained comparably narrowed to those post-procedure (long-term follow-up 6.7 (3.2–12.4) vs. post-procedure 5.6 (2.1–10.1), *p* = 0.368), rather than rebounding to levels similar to DBA_C_-pre (long-term follow-up 6.7 (3.2–12.4) vs. pre-procedure 11.1 (4.9–16.4), *p* = 0.021).

Furthermore, as depicted in Fig. 1B, when compared to the single stenting group, the dual stenting group exhibited a narrower DBA_C_-post (5.6 (2.1–10.1) vs. 7.5 (3.4–14.8), *p* = 0.002) and DBA_C_-long (6.7 (3.2–12.4) vs. 10.8 (5.2–16.9), *p* = 0.002), but with similar DBA_C_-pre (11.1 (4.9–16.4) vs. 11.2 (5.5–16.8), *p* = 0.462).

### 3.3 Lesion Progression 

**Fig. 3.**
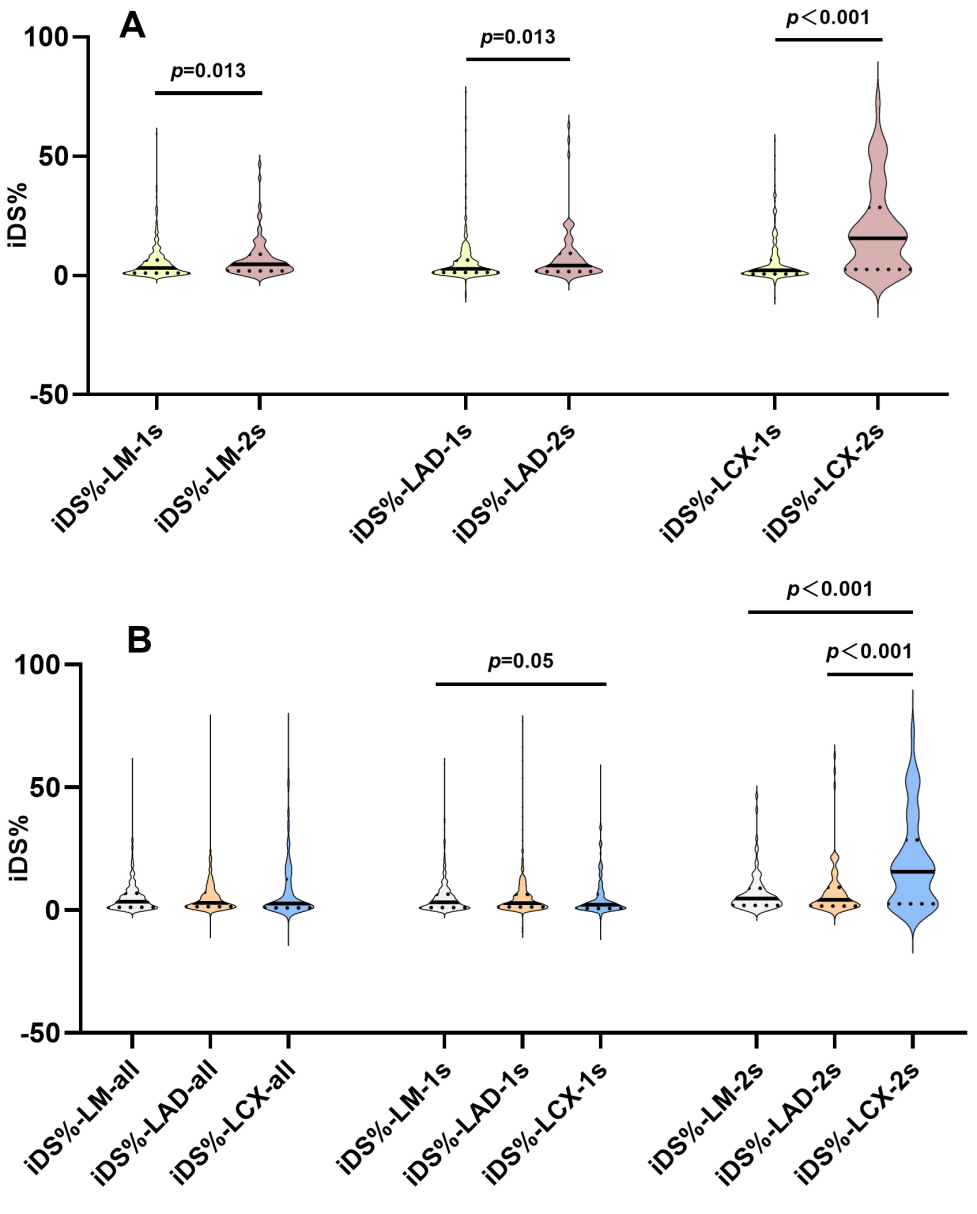
**iDS% across bifurcation segments by stenting strategy**. (A) Comparisons among iDS% in LM, LAD and LCX between the single and dual stenting groups. (B) Comparisons of iDS% in LM, LAD and LCX both in the single and dual stenting groups. iDS%, increase in percent diameter stenosis; LMCBL, left main coronary bifurcation lesion; LM, left main; LAD, left anterior descending; LCX, left circumflex; iDS%-LM-1s, iDS%-LAD-1s, and iDS%-LCX-1s, iDS% in LM, LAD and LCX in distal left main bifurcation patients treated with single stenting; iDS%-LM-2s, iDS%-LAD-2s, and iDS%-LCX-2s, iDS% in LM, LAD and LCX in distal left main bifurcation patients treated with dual stenting; iDS%-LM-all, iDS%-LAD-all, and iDS%-LCX-all, iDS% in LM, LAD and LCX in all included patients.

The QCA characteristics of LMCBL were presented in Tables 2,4. The dual stenting techniques were applied to patients with more complex bifurcation disease, particularly those with higher pre-procedural DS% in LM and LCX. However, the progression across the three segments of LMCBL following stenting was more pronounced in patients treated with dual stenting technique (Fig. 3A), especially in the LCX (single stenting: 2.2 (0.7–6.5) vs. dual stenting: 15.6 (2.5–28.6), *p* < 0.001).

**Table 4.** **Discrepancies in iDS% in LM, LAD and LCX**.

	LM	LAD	LCX	*p* value
iDS%-all	3.4 (1.2–6.9)	2.9 (1.3–7.3)	2.6 (0.9–12.6)	0.899
iDS%-single stenting	3.1 (1.1–6.5)	2.8 (1.2–6.5)	2.2 (0.7–6.5)	0.027
iDS%-dual stenting	4.7 (1.9–8.8)	4.2 (1.6–9.2)	15.6 (2.5–28.6)	<0.001

Values were median (interquartile range). iDS%, increase in percent diameter stenosis; LM, left main; LAD, left anterior descending; LCX, left circumflex; iDS%-all, iDS% in all included patients; iDS%-single stenting, iDS% in the single stenting group; iDS%-dual stenting, iDS% in the dual stenting group.

In the distinct individual iDS% across the three segments of LMCBL under the two interventional strategies, we also compared the differences of iDS% among the LM, LAD and LCX. As illustrated in Fig. 3B, in the single stenting group, the discrepancy was attributed to the lower iDS%-LCX compared to iDS%-LM (2.2 (0.7–6.5) vs. 3.1 (1.1–6.5), *p* = 0.05). But in the dual stenting group, the iDS%-LCX was significantly higher than both iDS%-LM (15.6 (2.5–28.6) vs.4.7 (1.9–8.8), *p* < 0.001) and iDS%-LAD (15.6 (2.5–28.6) vs.4.2 (1.6–9.5), *p* < 0.001).

### 3.4 Factors Associated With iDS%-LCX (Linear Regression)

Hierarchical multiple linear regression analysis (Table 5 and **Supplementary Fig. 1**) was conducted to explore the factors associated with iDS%-LCX (the dependent variable), which was prominent in the progression of LMCBL. Regression diagnostics confirmed that model assumptions were reasonably met. One outlier with a standardized residual slightly exceeding |3| was identified; however, its Cook’s Distance was well below 1, indicating it did not exert undue influence on the model’s overall stability or coefficient estimates. In Block 1, among the arteriosclerosis-related variables, the LCX stenosis immediate after the procedure (DS%-post in LCX, β = –0.287, *p* < 0.001) and dyslipidemia (β = –0.13, *p* = 0.024) were significantly associated with iDS%-LCX. The inclusion of dual stenting techniques (LMCBL with dual stenting) in Block 2 significantly improved the model, accounting for an additional 11.3% of the variance in iDS%-LCX (△R^2^ = 0.113, *p* < 0.001). When pre-, post-procedural PBA_C_ and DBA_C_ were entered in Block 3, they provided a modest but significant improvement to the model, with a further 1.3% increase in the explained variance (△R^2^ = 0.013, *p* = 0.208). In the final model, the dual stenting technique (LMCBL with dual stenting) exhibited the strongest association (β = 0.406, *p* < 0.001), followed by PBA_C_-pre (β = 0.109, *p* = 0.026). Taken together, these variables collectively explained 23.3% of the variance in iDS%-LCX.

**Table 5.** **Hierarchical multiple linear regression assessing predictions of iDS% in LCX**.

Variables predicting iDS%-LCX	B	SE	β	*t*	VIF	R	R^2^	ΔR^2^
Block 1						0.327	0.107	0.107^*⁣**^
						F = 3.554^*⁣**^		
Age	–0.086	0.073	–0.063	–1.167	1.142			
Male	–0.251	1.892	–0.007	–0.133	1.215			
Female (Ref.)								
Follow-up	0.002	0.001	0.058	1.131	1.032			
BMI	–0.118	0.239	–0.026	–0.493	1.111			
Diabetes mellitus	–0.519	1.450	–0.019	–0.358	1.082			
Hypertension	–1.453	1.495	–0.052	–0.972	1.123			
Current smoking	–0.010	1.545	0.000	–0.006	1.203			
Dyslipidemia	–3.618	1.600	–0.130	–2.261^*^	1.314			
LDL-C	0.291	0.712	0.023	0.409	1.257			
LVEF	0.004	0.063	0.004	0.071	1.035			
IVUS/OCT	0.048	1.467	0.002	0.033	1.030			
DS%-post in LCX	–0.304	0.057	–0.276	–5.349^*⁣**^	1.059			
Block 2						0.469	0.220	0.113^*⁣**^
						F = 7.684^*⁣**^		
Age	–0.122	0.069	–0.089	–1.778	1.148			
Male	0.326	1.772	0.010	0.184	1.218			
Female (Ref.)								
Follow-up	0.001	0.001	0.044	0.912	1.034			
BMI	–0.089	0.223	–0.020	–0.397	1.111			
Diabetes mellitus	–0.483	1.357	–0.017	–0.356	1.082			
Hypertension	–0.744	1.403	–0.026	–0.530	1.128			
Current smoking	–0.013	1.446	0.000	–0.009	1.203			
Dyslipidemia	–2.573	1.505	–0.092	–1.709	1.326			
LDL-C	0.036	0.668	0.003	0.055	1.261			
LVEF	–0.018	0.059	–0.015	–0.306	1.038			
IVUS/OCT	0.101	1.373	0.003	0.073	1.030			
DS%-post in LCX	–0.053	0.064	–0.048	–0.830	1.520			
LMCBL with single stenting (Ref.)								
LMCBL with dual stenting	13.028	1.794	0.409	7.156^*⁣**^	1.484			
Block 3						0.483	0.233	0.013
						F = 6.255^*⁣**^		
Age	–0.118	0.069	–0.086	–1.713	1.162			
Male	0.020	1.773	0.001	0.011	1.226			
Female (Ref.)								
Follow-up	0.001	0.001	0.050	1.036	1.046			
BMI	–0.085	0.224	–0.019	–0.378	1.123			
Diabetes mellitus	–0.584	1.357	–0.021	–0.430	1.087			
Hypertension	–0.774	1.409	–0.028	–0.549	1.143			
Current smoking	–0.168	1.456	–0.006	–0.115	1.227			
Dyslipidemia	–2.511	1.506	–0.090	–1.667	1.334			
LDL-C	0.103	0.669	0.008	0.153	1.274			
LVEF	–0.020	0.059	–0.016	–0.334	1.050			
IVUS/OCT	–0.143	1.377	–0.005	–0.104	1.043			
DS%-post in LCX	–0.051	0.064	–0.046	–0.795	1.528			
LMCBL with single stenting (Ref.)								
LMCBL with dual stenting	12.915	1.837	0.406	7.029^*⁣**^	1.521			
PBA_C_-pre	0.069	0.031	0.109	2.23^*^	1.089			
DBA_C_-pre	–0.066	0.080	–0.042	–0.823	1.159			
PBA_C_-post	–0.028	0.028	–0.048	–1.004	1.059			
DBA_C_-post	0.052	0.087	0.030	0.603	1.151			

Note: Dependent variables was Y. “*p < 0.05; ***p < 0.001” D-W = 1.865. The residual plot and the normal P-P plot shown in **Supplementary Fig. 1**. iDS%, increase in percent diameter stenosis; LCX, left circumflex; iDS%-LCX, iDS% in LCX; BMI, body mass index; LDL-C, low-density lipoprotein cholesterol; LVEF, left ventricular ejection fraction; IVUS/OCT, intravascular ultrasound/optical coherence tomography; DS%-post, percent diameter stenosis post procedure; LMCBL with single stenting, LMCBL patients treated with single stenting technique; LMCBL with dual stenting, LMCBL patients treated with dual stenting technique; PBA_C_/DBA_C_-pre and PBA_C_/DBA_C_-post, PBA_C_/DBA_C_ before and post procedure.

**Fig. 4.**
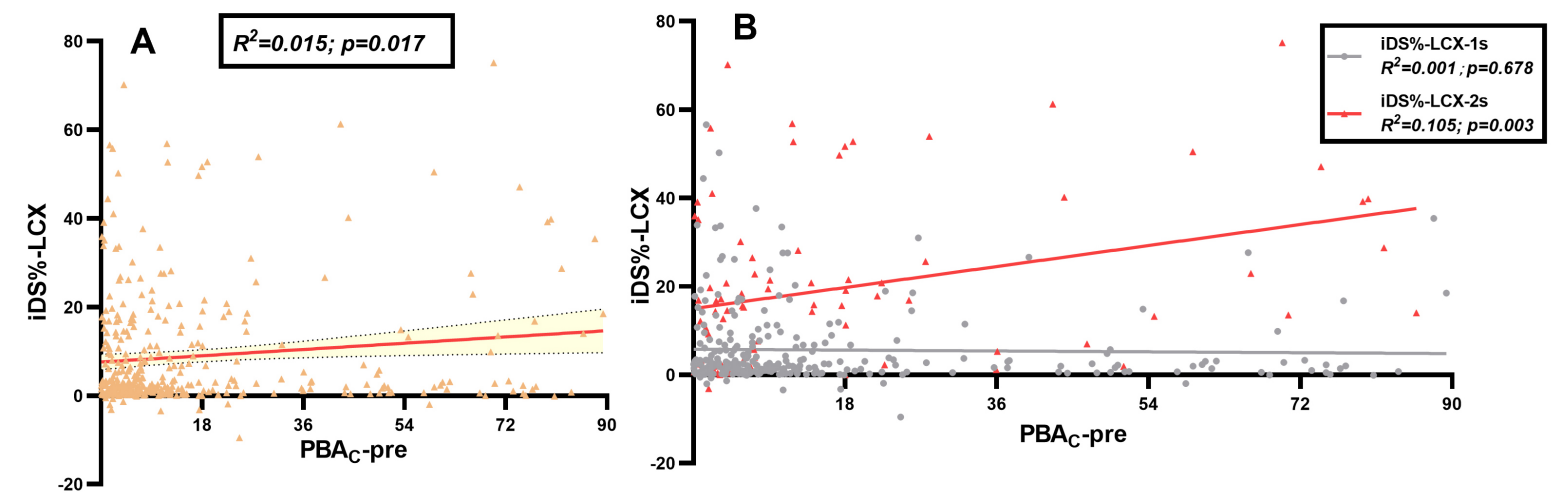
**Correlation of pre-procedural PBA_C_ with iDS% in LCX**. (A) Correlation of iDS% in LCX and pre-procedural PBA_C_ in all included patients. (B) Correlation of iDS% in LCX and pre-procedural PBA_C_, stratified by single and dual stenting. iDS%, increase in percent diameter stenosis; LCX, left circumflex; PBA_C_, proximal bifurcation angle change throughout the cardiac cycle; PBA_C_-pre, PBA_C_ before procedure; iDS%-LCX, iDS% in LCX; iDS%-LCX-1s, iDS% in LCX in distal left main bifurcation patients treated with single stenting; iDS%-LCX-2s, iDS% in LCX in distal left main bifurcation patients treated with dual stenting.

Moreover, as shown in Fig. 4A, the PBA_C_-pre exhibited a linear correlation with iDS%-LCX (R^2^ = 0.015, *p* = 0.017). Upon stratification by interventional strategies (Fig. 4B), it was observed that only the dual stenting technique exhibited a meaningful linear correlation (R^2^ = 0.105, *p* = 0.003). To assess the potential influence of sample size on our key finding, a post-hoc power analysis was performed. The result (power = 0.87) was statistically consistent with the observed significant *p*-value (*p* = 0.003), suggesting a low risk of a Type II error (i.e., missing an association of this magnitude) under the sample conditions of our study.

## 4. Discussion

Our study revealed several key findings: (1) In LMCBL patients, the temporal changes in PBA_C_ were similar across the pre-procedural, post-procedural, and long-term follow-up assessments. Moreover, PBA_C_ values did not differ significantly between patients who underwent single and dual stenting. (2) The DBA_C_ among LMCBL patients who underwent single stenting showed a tendency to narrow immediately post-stenting and subsequently widened over time to the level of before procedure. However, in LMCBL patients treated with dual stenting, the DBA_C_ remained at a reduced level during long-term follow-up, similar to that post-procedure. The extent of stent-induced DBA_C_ reduction in LMCBL was more pronounced in the dual stenting group. (3) The progressions of lesions following stenting were notably pronounced in LMCBL patients subjected to dual stenting technique, especially in the LCX. (4) The pre-procedural PBA_C_ was identified as an independent anatomical risk marker of future LCX progression in LMCBL patients treated with dual stenting technique.

### 4.1 Temporal Changes in BA_C_

Previous studies revealed that stent implantation in LMCBL could decrease the BA cyclic range, especially with the dual stenting technique. In the substudy of the SYNTAX trial, Girasis *et al*. [13] found that the diastolic DBA and DBA range through the cardiac cycle had decreased following stenting. Watanabe *et al*. [7, 11] had also found this trend in complex dual stenting. In the studies by Wang *et al*. [8], the restriction of cyclic angulation range for both BA_L⁢M-L⁢C⁢X_ (defined as 180^∘^-PBA) and DBA had been detected among patients undergoing dual stenting. Our study confirmed the findings of prior research regarding DBA_C_, documenting a narrow tendency in LMCBL patients regardless of whether they were treated with the single or dual stenting. Furthermore, a more marked reduction in DBA_C_ post-stenting was observed in LMCBL patients who received dual stenting compared to those undergoing single stenting. However, DBA_C_ values were different during long-term follow-up, with a rebound to pre-procedural levels in the single stenting group and remaining at post-procedural levels in the dual stenting group.

The decrease in DBA_C_ following stent implantation was attributed to the longitudinal straightening effect, which aligned with the myocardial motion during systole and contrasted during diastole, resulting in a reduction of the DBA throughout the cardiac cycle post-stent implantation [8]. However, in our study, we found that the most significant impact on DBA_C_ was the side branch stenting, which not only caused a more significant decrease in the range of DBA_C_, but also disrupted the reverting effect during the long-term follow-up. The phenomenon of the reverting effect in DBA_C_ following stenting occurred as the epicardial coronary arteries attempted to return to their original geometry by exerting periodic, repetitive strain on the metallic stent. Stent endothelialization and compression, or even fracture at the stent hinge point, might play a critical role in the process of reverting the effect in DBA_C_. However, in the dual stenting technique, the reverting effect was compromised due to the restriction of the side branch throughout the cardiac motion, and the change in bifurcation geometric shape was more pronounced. The excessive metal stent struts in the bifurcation core area might be the key factor preventing the bifurcation geometry from reverting to its preoperative state.

Conversely, the longitudinal straightening effect following stenting and the reverting effect over the long term were not statistically significant for PBA_C_. The temporal changes in PBA_C_ differed from those of DBA_C_. At pre-procedural, post-procedural, and long-term follow-up assessments, values were similar in LMCBL patients regardless of whether they were treated with single or dual stenting. No difference was detected between the two interventional strategies. The distinction between DBA_C_ and PBA_C_ might be clarified by Torrent-Guasp’s spiral myocardial band theory [15, 16]. This theory suggested that myocardial contraction and relaxation did not occur as the inflation and deflation of a balloon. Instead, they originated at the base of the heart and propagated along the myocardial band, causing various parts of the heart to contract sequentially, similar to “twisting a towel in a spiral”. The near-multiple difference in the thickness of the left and right ventricular walls, resulting from the myocardial band wrapping around either once or twice, led to uneven strain on the PBA_C_ and DBA_C_ in LMCBL. The stent implantation, including dual stents, was unable to counteract the robust strain of myocardial contraction and relaxation, which might explain why PBA_C_ maintains a comparable effect over time.

### 4.2 Impact of BA_C_ on iDS%

In our study, when compared to the single stenting group, the dual stenting group exhibited more severe lesion progression in the LM, LAD, and LCX. Furthermore, the iDS% in LCX was far exceeded those in the LM and LAD. The fastest progression for the single stenting group occurred in the LM, not in the LCX. These findings might indicate that the stent itself might serve as a predictor of lesion progression [17].

Regarding BA_C_, previous studies had failed to reach a consensus on clinical adverse events. Some suggested that pre-procedural DBA_C_ contributed to the target lesion failure in LMCBL, others supported post-procedural DBA_C_, and still others believed that PBA_C_ also played an important role. The hypothesis was that a larger BA_C_ served as a surrogate for the greater hinge motion of coronary arteries, moving in step with cardiac motion. The implanted stents in LMCBL with larger BA_C_ were exposed to more compression, torsion, kinking, elongation, bending, and shear stress due to cardiac contractions, which were associated with stent-related adverse events [7, 18].

When compared to the single stenting group, the extent of decreased DBA_C_ post-stenting was more prominent in the dual stenting group, mainly driven by the multiple overlaps of metal stent struts at the ostium of side branches. The excessive overlap of metal stent struts in bifurcation core area was usually linked to clinical adverse events [19, 20]. Therefore, to some extent, the reported correlation between DBA_C_ and target lesion failure could be transformed into the relationship between the overlap extent of metal stent struts in dual stenting approach and lesion progression.

However, in our study, except for pre-procedural PBA_C_ in patients using the dual stenting, the DBA_C_ was not associated with lesion progression, regardless of whether it was performed with a single or dual stenting technique. This seemed to contradict the aforementioned studies. The endpoints selected in our study were angiographic lesion progression, as assessed by QCA, whereas other studies chose target lesion failure or revascularization as their endpoints. The degree of lesion progression in other studies was much greater than in our study, which might be why previous studies were able to achieve positive results with a comparable sample size.

Our analysis identified a significant yet modest association between pre-procedural PBA_C_ and lesion progression (iDS%-LCX) in the dual stenting group, accounting for approximately 10.5% of the variance. This finding aligned with the insights from Wang *et al*. [8], reinforcing the role of bifurcation anatomy in mechanistic environmental perturbations. Furthermore, the hierarchical regression demonstrated that pre-procedural PBA_C_ provided independent predictive value beyond traditional atherosclerotic risk factors, which were not consistently correlated with progression in our model. This suggested that mechanical factors inherent to the bifurcation, partly captured by PBA_C_, played a distinct role. However, the mechanistic interpretation of these findings was limited by the absence of intracoronary imaging (IVUS/OCT), which precluded definitive insights into underlying factors such as stent expansion, apposition, or the extent of strut overlap [21, 22].

The identification of pre-procedural PBA_C_ as an independent anatomical risk marker opened new avenues for future investigation. Prospective studies should aim to validate this association and explore whether combining pre-procedural PBA_C_ with hemodynamic metrics (e.g., FFR), biomarkers, or advanced imaging modalities (e.g., IVUS/OCT) could lead to the development of a risk stratification model with greater predictive power (higher R^2^) and clinical utility. In this context, integrating PBA_C_ assessment with intracoronary imaging represented a particularly promising path. Future studies could investigate the synergy whereby baseline PBA_C_ informed anatomical planning, while IVUS/OCT provided direct verification of optimal stent deployment [23, 24], potentially enhancing the paradigm for optimizing left main bifurcation intervention.

### 4.3 Limitation 

This study had several limitations. First, it was retrospective and observational, with a limited sample size. Second, while selection bias due to the requirement for complete angiographic follow-up limited the external validity of our findings, it did not logically break the mechanistic link between variables within the studied cohort. Therefore, the internal validity of the association between pre-procedural PBA_C_ and iDS%-LCX remained robust. Third, it was unclear whether the angiographic follow-up was routine or symptom-directed. However, our study detailed the angiographic restenosis in the LMCBL, which would more clearly exhibit lesion progression, rather than symptom-directed angiographic follow-up potentially driven by other vessels outside the LMCBL. Fourth, our study concentrated exclusively on the progression of lesions within three segments of the LMCBL, not investigating those outside the LMCBL. Furthermore, the correlations between BA_C_ and clinical outcomes were not evaluated in our study. Fifth, the low utilization rate of intracoronary imaging devices meant that IVUS/OCT data were not obtained, which precluded a more mechanistic interpretation of the results. The potential impact of detailed interventional strategies should be specifically clarified in future studies, such as with or without a branch ostial optimization technique in the single stenting group, as well as provisional T, Culotte, and Crush techniques in the dual stenting group. Finally, a direct comparison between alternative techniques for assessing the cyclic BA range in LMCBL was not performed. This included, for instance, a comparison of 3D reconstruction against 2D consistent optimal view measurements across the pre-procedural, post-procedural, and long-term follow-up phases. Further studies are needed to explore the mechanism behind changes in BA_C_, thereby elucidating the correlation between BA_C_ changes and lesion progression or adverse clinical events. In vitro dynamic bench tests and the application of intracoronary imaging devices could help to understand the relation between BA_C_ changes and the overlap extent of metal stent struts in bifurcation core area.

## 5. Conclusions

The PBA_C_ in LMCBL remained unaltered by interventional strategies and over time, whereas the DBA_C_ significantly decreased immediately following stenting, particularly in the dual stenting approach. However, during long-term follow-up, it rebounded to pre-procedural levels in the single stenting group and remained at post-procedural levels in the dual stenting group. The pre-procedural PBA_C_ emerged as an independent anatomical risk marker for lesion progression in the LCX following dual stenting. This exploratory finding warrants future prospective validation and may open new avenues for research into anatomical risk stratification.

## Data Availability

All data reported in this paper are available from the corresponding author on reasonable request.

## Abbreviations

PCI, Percutaneous coronary intervention; LMCBL, left main coronary bifurcation lesion; CABG, coronary artery bypass grafting; BA, bifurcation angle; LAD, left anterior descending; LCX, left circumflex; BA_C_, BA change throughout cardiac cycle; LM, left main; 3D, three-dimensional; 2D, two-dimensional; QCA, quantitative coronary angiography; PBA, proximal bifurcation angle; DBA, distal bifurcation angle; PBA_C_, PBA change throughout the cardiac cycle; DBA_C_, DBA change throughout the cardiac cycle; DS%, percent diameter stenosis; iDS%, increase in DS%; ICC, Intraclass Correlation Coefficient; BMI, body mass index; LVEF, left ventricular ejection fraction; LDL-C, low-density lipoprotein cholesterol; IVUS, intravascular ultrasound; OCT, optical coherence tomography; PBA_C_-pre, PBA_C_ before procedure; PBA_C_-post, PBA_C_ post-procedure; PBA_C_-long, PBA_C_ during long-term follow-up; DBA_C_-pre, DBA_C_ before procedure; DBA_C_-post, DBA_C_ post-procedure; DBA_C_-long, DBA_C_ during long-term follow-up; iDS%-LM, iDS% in LM; iDS%-LAD, iDS% in LAD; iDS%-LCX, iDS% in LCX.
