# Supplementary material for: Long-Term Lesion Progression After Left Main Distal Bifurcation Stenting: Insights From Bifurcation Angle Variation Throughout the Cardiac Cycle
Source: Rev Cardiovasc Med. 2026 May 13;27(5):45495. doi: 10.31083/RCM45495 (PMC13227362; doi:10.31083/RCM45495)
Supplement: Supplementary file 1 [file 2153-8174-27-5-45495-s1.zip › Supplementary Material.docx]

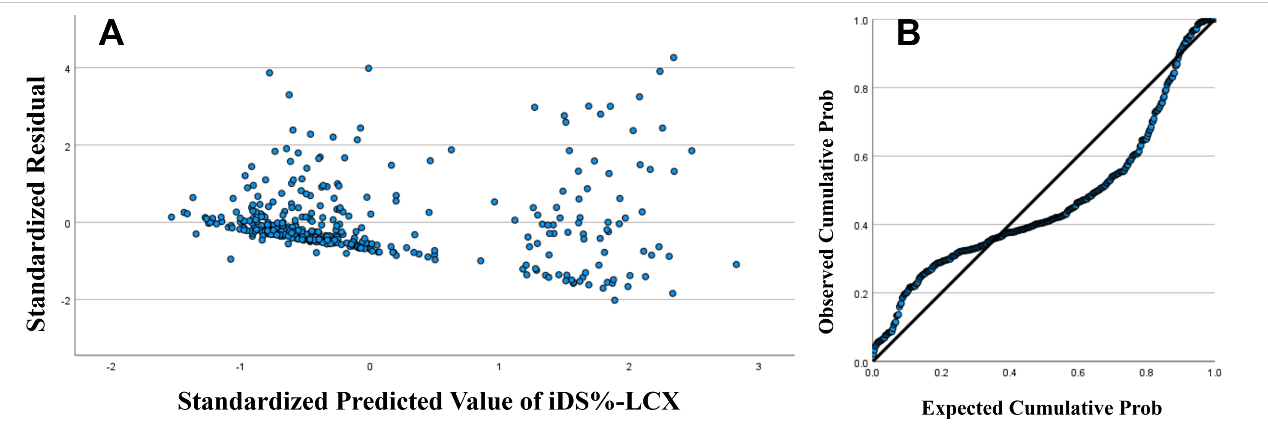


**Supplementary Fig. 1.** (A) Residual plot from the hierarchical multiple linear regression analysis. (B) Normal P-P plot from the hierarchical multiple linear regression analysis.
